# Synthesis and Characterization of Paclitaxel-Loaded PEGylated Liposomes by the Microfluidics Method

**DOI:** 10.1021/acs.molpharmaceut.3c00596

**Published:** 2023-11-06

**Authors:** Eman Jaradat, Edward Weaver, Adam Meziane, Dimitrios A. Lamprou

**Affiliations:** † School of Pharmacy, Queen’s University Belfast, 97 Lisburn Road, BT9 7BL Belfast, U.K.; ‡ Fluigent, 94270 Le Kremlin-Bicêtre, France

**Keywords:** liposomes, paclitaxel, nanomedicine, microfluidics, chemotherapy, cancer

## Abstract

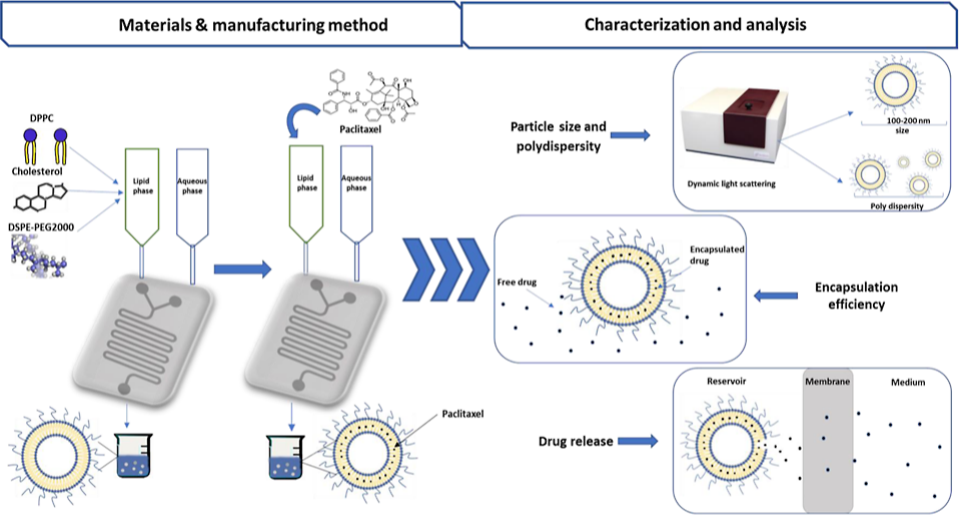

For cancer therapy, paclitaxel (PX) possesses several limitations, including limited solubility and untargeted effects. Loading PX into nanoliposomes to enhance PX solubility and target their delivery as a drug delivery system has the potential to overcome these limitations. Over the other conventional method to prepare liposomes, a microfluidic system is used to formulate PX-loaded PEGylated liposomes. The impact of changing the flow rate ratio (FRR) between the aqueous and lipid phases on the particle size and polydispersity index (PDI) is investigated. Moreover, the effect of changing the polyethylene glycol (PEG) lipid ratio on the particle size, PDI, stability, encapsulation efficiency % (EE %), and release profile is studied. The physicochemical characteristics of the obtained formulation were analyzed by dynamic light scattering, FTIR spectroscopy, and AFM. This work aims to use microfluidic technology to produce PEGylated PX-loaded liposomes with a diameter of <200 nm, low PDI < 0.25 high homogeneity, and viable 28 day stability. The results show a significant impact of FRR and PEG lipid ratio on the empty liposomes’ physicochemical characteristics. Among the prepared formulations, two formulations produce size-controlled, low PDI, and stable liposomes, which make them preferable for PX encapsulation. The average EE % was >90% for both formulations, and the variation in the PEG lipid ratio affected the EE % slightly; a high packing for PX was reported at different drug concentrations. A variation in the release profiles was notified for the different PEG lipid ratios.

## 1 Introduction

Over recent years, significant efforts have focused on improving the therapeutic efficacy and safety of cancer treatments. The various existing traditional cancer treatments, including surgery, radiotherapy, and chemotherapy, are the current gold standard in clinical studies and practices. Chemotherapy is the conventional type of cancer treatment used, which destroys the malignant cancerous cells and limits their metastasis to other tissues by inhibiting different phases of the cell division process.^1^ The limitation of using chemotherapies is highlighted by the active pharmaceutical ingredient’s (API) high plasma concentration, which leads to high toxicity and severe side effects. Also, the random and uncontrolled distribution of chemotherapies causes undesirable effects on healthy tissues and destroys human immunity.^2^

Paclitaxel (PX) is one of the most significant chemotherapies on the market today. The success of PX is owed to its properties, including the antitumor activity over a wide range of cancers, the ability to attack solid and disseminated tumors, and the inspiring mechanism of action. PX works as a microtubule-stabilizing API that disrupts microtubule movement. This consequently arrests the cell cycle and causes cell death. PX can be used widely to treat a broad spectrum of cancers, including metastatic breast cancer, nonsmall cell lung cancer, and ovarian cancer.^3^ The main drawback of using PX is due to the low aqueous solubility (less than 0.1 mg/mL) of the drug in the aqueous solvent, which impedes the formulation of the drug as an intravenous formulation. Since the early years, researchers have made major efforts in past research to increase the PX solubility by different techniques, such as adding charged agents to PX formulations or formulating as a salt form, which was not feasible for PX.^4,5^ Other studies tried to formulate PX as a prodrug; for example, Surapaneni et al. tried to formulate the prodrug by substituting the 2′ position as the optimum position, and the result shows rapid hydrolysis in vivo into 2′-acyl-PX derivatives in the blood.^5^ Nicolaou et al. performed esterification to formulate a PX ester substituted with a strong electron-withdrawing agent, such as an alkoxy group, to accelerate the hydrolytic cleavage.^6^ The in vitro studies of the prodrugs show cytotoxic effects on the cancerous cells comparable to those of the conventional PX. Moreover, other efforts worked on changing pH.^5^ This was relatively unsuccessful, though, as the chemical structure of PX lacks any ionizable groups within the pharmaceutically active range, which makes any pH alterations ineffective for enhancing the solubility.^7^ As detailed, none of the aforementioned efforts overcame the untargeted and ineffective delivery of PX, which results in harmful effects on healthy cells and organs.

Lately, the development of biomedical nanotechnology for targeting API delivery is one of the innovative techniques that has enhanced the therapeutic effect of chemotherapy.^8^ Nanoparticles (NPs) have the potential to overcome the limitations of conventional chemotherapy, such as insufficient efficacy, poor biodistribution, lack of sensitivity, and toxicity. Targeting the drug delivery of chemotherapies provides multiple advantages over using conventional medicines, such as improving the bioactive performance of the drugs, overcoming the dilemmas of drug resistance, and diminishing the drug toxicity to healthy physiological tissues. Loading chemotherapeutic APIs into nanocarriers to target their delivery shows promising results in reducing the toxic effects on healthy tissues and preventing any immunological responses.^9^ For example, NPs show success in encapsulating PX and enhance their pharmacodynamics and safety. Pazenir is a PX albumin-bound NP that was approved to be in the market in 2019 after displaying clinical efficacy and safety.

Among the different nanocarriers, lipid nanocarriers, liposomes specifically, possess the lowest toxicity of most common nanocarriers, with the potential to encapsulate both hydrophobic and hydrophilic molecules.^10,11^ Moreover, liposomes have the ability to act as a solubilizing agent for low-solubility drugs by encapsulating them within the lipid bilayer.^12^ This makes liposomes one of the most promising nanocarriers since 40% of chemical entities have low aqueous solubility.^13^ Several studies in the literature highlight the positive impact of encapsulating cancer drugs into liposomes, such as improving the therapeutic index, increasing the uptake by tumor cells, and inhibiting tumor cell growth.^14,15^ Moreover, liposomes can effectively target cancer drug delivery by active or passive targeting. Passive drug delivery targeting relies on the enhanced permeability and retention (EPR) effect. The EPR effect can be explained by the rapid proliferation of tumors, which results in neovascularization of the cancerous tissue, which is characterized by large fenestrations and limited lymphatic drainage. This unique vascular structure activates the EPR effect and enables the liposomes to pass through the relatively permeable blood vessels within the tumor and accumulate at the desired location. In order to enhance the localization and accumulation of the liposomal cancer drugs and avoid healthy cell affection, active targeting liposomes developed. Active drug delivery targeting can be achieved by targeting the overexpressed receptors on the surface of cancerous cells or targeting the cancer tissue’s microenvironment. Different ligands can attach to liposome surfaces for active targeting, including proteins, antibodies, and peptides. In general, two approaches can be used to functionalize the surface of liposomes with specific legends. The first approach involves attaching the targeting ligand to one lipid and mixing it with other lipids to formulate liposomes. This approach is inconvenient to use; attaching large-size ligands to a lipid complicates the process and affects the ligands’ efficacy due to the multiple exposures to organic solvents. Alternatively, liposome functionalization with ligands is performed for preformulated liposomes by attaching ligands to the liposome surface. For this approach, specific lipids modified with polyethylene glycol (PEG) spacer and amine-functionalized carboxylic acid, thiol, or maleimide groups are mixed with the other lipids to formulate the liposome. Incorporating PEGylated lipids in the liposome composition offers excellent opportunities to attach the ligands on the surface of liposomes by forming chemical bonds (amide conjugation, hydrazone bond, thioester, or disulfide bridge formation). Also, using PEGylated lipids decreases the required amounts of targeting ligands, which will facilitate the binding of large molecules such as proteins.

However, a significant improvement in nanoliposomes has arisen after incorporating PEG within the nanovessels, causing a modification of the surface of liposomes (stealth liposomes). PEG is a neutral, thermoplastic, and crystalline copolymer characterized by low toxicity and immunogenicity and high biocompatibility, and the FDA has approved it for pharmaceutical formulations.^16^ The PEGylation of liposomes results in increasing their stability as a drug delivery system (DDS) by providing steric stabilization, preventing aggregation, extending liposomes’ half-life in blood circulation, and avoiding the uptake by the reticuloendothelial system.^17^ PEG-lipid chains provide a more hydrated and hydrophilic form of the liposomal surface, which can limit the protein adsorption and opsonization of liposomes. This will give the PEGylated liposomes (PEG liposomes) the ability to pass through the liver and spleen without any clearance and extend the duration of drug exposure to tumor tissue due to depleted lymphatic drainage.^18^ The studies reported a major impact on the physicochemical properties of liposomes after PEG incorporation, specifically the particle size and polydispersity.^19,20^ Moreover, the PEGylation of liposomes can impact the encapsulation efficiency (EE %), tissue distribution, and in vivo release.^21^

It is well-known that the physical properties of liposomes do not rely on the lipid composition only; the liposome’s preparation method is one of the significant parameters that affect liposomal size, polydispersity index (PDI), and lamellarity. Several traditional methods have been used over the years to fabricate PEG liposomes, including thin-film hydration and extrusion. Significant limitations have been reported for both methods, such as being a time-consuming multistep procedure with high batch-to-batch variation and scaling-up difficulties. Recently, hydrodynamic microfluidics (MF) has been utilized in manufacturing PEG liposomes. MF is an innovative technique that manipulates a small volume of fluids (10–9 to 10–18 L) using micrometer channels, microvalves, and micromixers as an interconnected system. The MF system offers a continuous laminar flow; this type of flow offers a high-quality mixing for the liposomal formulations, improving the size control, and homogeneity.^11^ Also, the enhancement of mixing quality relies on the capability to control the flow rate ratios (FRR) and total flow ratio (TFR) of the lipid and aqueous phases, which allows for the continuous production of monodisperse and homogeneous liposomes. The changes in TFR and FFR present an apparent effect on the particle size and PDI of the formulation; determining the optimum FRR and TFR is critical in producing well-formulated PEG liposomes. Several studies reported the significance of determining the suitable TFR and FRR in improving liposome size, PDI, EE %, and stability.^22,23^ This work highlights the impact of changing the lipid composition, specifically PEG lipid ratios, as well as the FRR on the PEG liposome size, PDI, EE %, stability, and release profile.

## 2 Materials and Methods

### 2.1 Materials

**Figure 1:**
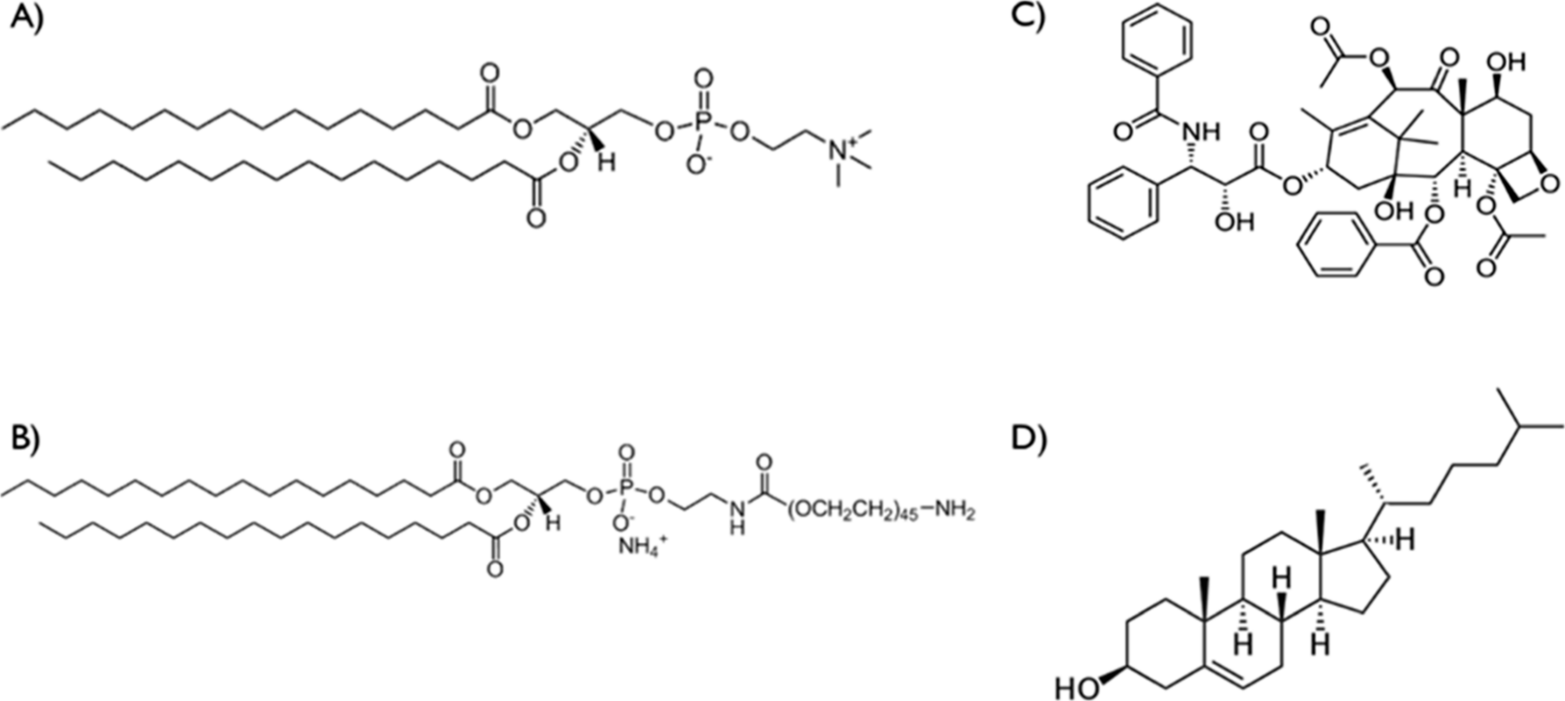
Chemical structure of (A) DPPC, (B) DSPE-PEG200, (C) PX, and (D) cholesterol.

1,2-Dipalmitoyl-*sn*-glycero-3-phosphocholine (DPPC) was purchased from TCI. 1,2-dipalmitoyl-*sn*-glycero-3-phosphoethanolamine-*N*-[methoxy(polyethylene glycol)-2000] (DSPE-PEG200) (ammonium salt) was purchased from Avanti polar lipids. Cholesterol, tablets of phosphate-buffered saline (PBS, pH 7.4), Tween 80, ethanol ≥99.8%, and PX were all purchased from Sigma-Aldrich. Acetonitrile ≥99.9% was purchased from Honeywell. The chemical structures can be seen in Figure 1.

### 2.2 Methods

#### 2.2.1 Liposome Preparation

The PEG liposomes were prepared using a FLUIGENT MFCS-EZ (Paris, France) microfluidic flow control device and software. For empty PEG liposome preparation, the DPPC, cholesterol, and PEG lipid combined in five different mass ratios as presented in Table 1.

**Table 1:** Mass and Molar Ratios of the Formulations

formulation code	phospholipid/cholesterol/DSPE-PEG2000 mass ratio	phospholipid/cholesterol/DSPE-PEG2000 molar ratio
P22	2:2:2	0.32:0.6:0.08
P29	2:0.9:0.1	0.58:0.41:0.02
P27	2:0.75:0.25	0.57:0.41:0.02
P19	1:0.9:0.1	0.37:0.62:0.01
P17	1:0.75:0.25	0.49:0.5:0.013

The calculated masses of lipids are weighed and dissolved in a specific volume of ethanol (≥99.8% v/v) and then sonicated to prepare the lipid phase with a total lipid concentration of 1 mg/mL.^23,24^ The sonication step is essential to ensure complete dissolution of the lipids. The prepared lipid phase is inserted into the first chamber, and the PBS (pH 7.4) is inserted into the second chamber as the aqueous phase. The lipid and aqueous phases are injected into a Y-shaped MF chip with a 100 μm channel d diameter. The two phases are injected with three different ethanols to PBS FRR 1:4, 1:5, 1:6, and TFR 1 mg/mL.

For loaded PEG liposomes, the most suitable FRR and the most stable formulations from the empty liposome studies were used to encapsulate the PXT. PXT is dissolved in the lipid phase at two different concentrations of 0.08 mg/mL (1:12 drug-to-lipid ratio) and 0.1 mg/mL (1:10 drug-to-lipid ratio). The reason for using these concentrations is discussed in Section 3.2. Every formulation is prepared nine times to allow for statistical analysis and reproducibility data.

#### 2.2.2 Particle Sizing and ζ-Potential

The particle size and PDI were measured by dynamic light scattering (DLS) using the Nanobrook Omni particle sizer (Brookhaven Instruments, Holtsville, NY, USA). Twenty μL portion of the liposomal formulation was diluted with PBS up to 2 mL. The same system was also used to measure the ζ-potential. Each sample was measured three times, again using samples that were originally produced in triplicate.

#### 2.2.3 Fourier Transform Infrared Spectroscopy

FTIR analysis was performed for the PEG liposomes using an attenuated total reflection (ATR)-FTIR spectrometer (Thermo Fisher Scientific, Nicolet is 50 FTIR with built-in ATR) to study the impact of modifying liposomes surface with PEGylated lipid on the stretching or bending of chemical bonds or generating new bonds. The samples were prepared by centrifuging the liposome formulations at 14,800 rpm for 30 min, then collecting the remaining pellets for analysis. The liposome suspensions were examined in an inert atmosphere over a wave range of 4000–600 cm^–1^ over 64 scans at a resolution of 4 cm^–1^ and an interval of 1 cm^–1^. Every sample was tested three times, and all the samples were tested on day 0 to decrease the incidence of formulation degradation.

#### 2.2.4 Atomic Force Microscopy

The AFM TT-2 AFM (AFMWorkshop, US) was used to study the morphology of the PEG liposomes. Twenty μL of each sample is diluted up to 2 mL of PBS water, then 20 μL of the solution is placed on a cleaved mica surface (1.5 cm × 1.5 cm; G250–2 Mica sheets 1 in. × 1 in. × 0.006 in.; Agar Scientific ltd., Essex, UK) and left to dry for 30 min. Then, samples were subject to a sheer wash with 1 mL of PBS to remove any nonadhered liposomes from the mica surface. Again, the solution was left to dry for 30 min before scanning. The AFM images were achieved by Ohm-cm Antimony doped Si probes, with a frequency range of 50–100 kHz. AFM images were performed at a resolution of 512 × 512 pixels at a scan rate of 0.6 Hz.

#### 2.2.5 Stability Studies

The stability study proceeded at two different temperatures to study the particles’ stability and compatibility at storage conditions (4 °C) and body temperature (37 °C) to mimic the conditions during the shelf life and inside the body after administration. Every sample proceeded to three analyses, and the samples were divided into two groups, and every group was stored at 4 and 37 °C. The size, PDI, and ζ-potential were tested weekly for up to 4 weeks. The analysis was performed for the different ratios of PEGylated liposomes to determine the most stable lipid-to-PEG lipid ratio. Formulations were stored as liquid suspensions and set in place to determine the physical stability of the formulations over the storage period.

#### 2.2.6 Encapsulation Efficiency

The dialysis method is used to eliminate the unentrapped drug from the formulations. The dialysis bags (cellulose membrane, avg flat width 10 mm, 0.4 in., MWCO 14,000, Sigma-Aldrich) were boiled in deionized water (DI) and then rinsed with DI water to sterilize the bags prior to analysis. The 14 kDa (14,000 g/mol) membrane with an estimated 2–3 nm pore size was selected due to the molecular weight of PX (853.9 g/mol) and the PX-loaded liposomes’ diameter, which has a minimum diameter of approximately 20 nm. This membrane facilitates the free diffusion of PX through the membrane to the external medium while concurrently trapping the loaded liposomes within the internal medium.^25^ The prepared liposomal formulation was added to the bags and transferred into PBS with 2% Tween for 12 h at room temperature. Tween 80 is used in the external medium to enhance PX dissolution.^25,26^ The samples were withdrawn from the medium at 1,3,6,9, and 12 h, and the API content was analyzed by ultraviolet high-performance liquid chromatography.^27^ The free API is measured using a C18 column (250 × 4.6 mm) from Thermo Fisher Scientific at 227 nm. Acetonitrile and water were used as the mobile phases with a 50:50 gradient and isocratic elution flow. The sample injection volume was 50 μL, and the total flow rate was 1 mL/min for 10 min. The used method is validated upon PXT encapsulated liposomes in the literature.^25,28,29^Equation 1 is used for calculating the EE %.
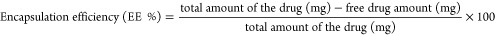
1

#### 2.2.7 In Vitro Drug Release Study

The dialysis tubing method was used to study the in vitro PXT release profile from the PEG liposomes. The dialysis bags (cellulose membrane, avg flat width 10 mm, 0.4 in., MWCO 14,000, Sigma-Aldrich) have been sterilized prior to analysis by boiling them in DI water and rinsing them with DI water. The preparation of the samples started with centrifuging the liposomal formulation for 30 min at 14,800 rpm. The resultant supernatant is withdrawn, and participant liposomal pellets are hydrated with PBS water and added to the dialysis bags. The dialysis bags were immersed in a release medium consisting of PBS water with 3% Tween 80, then transferred to a 37 °C incubator to initiate the release study. The release medium of PBS buffer is prepared to mimic the in vivo environment with pH 7.4.^30,31^ Tween 80 has been added to the release medium to achieve sink conditions by enhancing the ability to dissolve the released PX.

#### 2.2.8 Statistical Analysis

All experiments were performed in triplicate trials, and standard deviation and mean were calculated when required. One-way ANOVA tests have been performed for empty P29, P22, P27, P17, and P19.

## 3 Results and Discussion

### 3.1 Optimization of Empty PEGylated Liposomal Formulation

**Figure 2:**
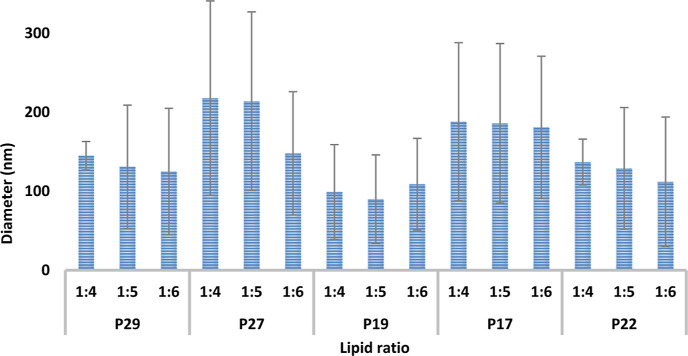
Average diameter of the empty PEG liposomes at different FRRs.

This work aims to use microfluidic technology to produce PEGylated PX-loaded liposomes with a diameter of <200 nm, low PDI < 0.25, high homogeneity, and viable 28 day stability. Multiple formulation parameters have been studied, specifically using different PEG lipids ratios and changing the FRR to achieve the optimum formulation. The powerful role of MF is the ability to control the different parameters of the manufacturing method, such as the FRR, that can highly affect the liposome’s diameter and homogeneity.^22,32^ Different FRRs have been investigated with every lipid ratio to study the impact of changing the FRR on the liposome diameter and PDI. As seen in Figure 2, the three FRRs of 1:4, 1:5, and 1:6 (lipid/aqueous) show an inverse relationship between the FRR increasing and the diameter of the liposome decreasing. Increasing the FRR from 1:4 to 1:5 to 1:6 represents a remarkable decrease in the liposome’s diameter (153 ± 19), (147 ± 70), and (127 ± 76), respectively. The reported result supports the trend of our previous work^22^ and other studies in the literature.^33,34^ Although the particle size decreased with increasing FRR, growth in the PDI values can be noticed. The PDI values and SD of 1:5 and 1:6 FRR were high, which indicates a lack of homogeneity and unreproducible liposomes. The particle size change by increasing the FRR can be explained by understanding the mixing process conditions; liposome creation mainly occurs due to the self-assembly of the lipids after mixing with an aqueous solution, also known as nucleation. The increase of the FRR reduces the solvent’s final concentration, which will keep the liposomes at their original diameter after nucleation and decrease the incidence of particle infusion, also known as the Ostwald ripening phenomenon.^12,35^ Decreased incidence of Ostwald ripening when increasing the FRR can explain the decrease of the liposome’s sizes. At the same time, the FRR ratio is proportional to the lipid concentration; for example, the same lipid concentration and experimental conditions were used in our previous work to produce conventional liposomes with 1:2, 1:3, and 1:4 FRRs; the particle size and PDI decreased when the FRR increased, which supports the aforementioned explanation. In this work, the growth of the PDI values (Figure 2) and the lack of homogeneity when the FRR increased from 1:4 to 1:5 to 1:6 is highly evident. This can be explained by the relevance between the dilution factor of the lipid phase and FRR; decreasing the lipid concentration at higher FRR reduces the diffusion rate, which leads to partially incomplete nucleation and variation between rates of liposome formation.^12^ Studying multiple FRRs can optimize the formulation method to achieve the optimum mixing and resultant liposomes as a result.

**Figure 3:**
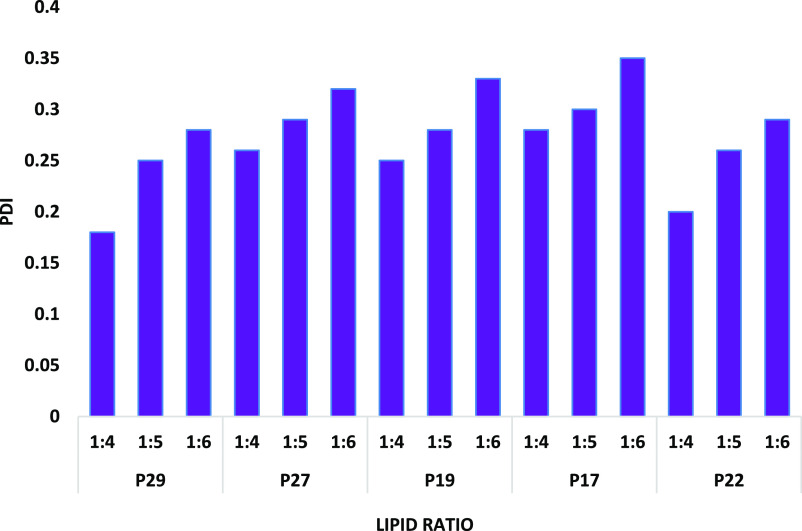
Average PDI values for empty PEG liposomes at different FRRs.

Furthermore, the study focused on the impact of varying the lipids to PEG lipid ratios on the liposomes̀ diameter, PDI, stability, EE %, drug loading, and release profile. The Incorporation of the PEGylated lipid into the liposome’s composition with an appropriate ratio offers a steric stabilization effect for the liposome.^36^ The results highlighted the major impact of the ratio between the lipids and PEG lipids on the stability and reproducibility of the liposomal formulation. The results (Figure 2) show that coupling the PEG lipids with DPPC and cholesterol as P29 and P22 ratio results in liposomal formulation with suitable diameter, high stability, and reproducibility. Compared to other different ratios, P27, P19, and P17, the resultant formulation shows a very high SD between the particle sizes from day 0, unreproducible formulation, and lack of stability. One-way ANOVA tests were performed for the empty PEG liposomal results, identifying *P* < 0.001, which confirms a significant difference between the ratios. In general, modifying conventional liposomes with PEG lipids has a major effect on the physiochemical characteristics of liposomes, specifically the liposomal diameter. For example, the reported results of our previous work^22^ (results not shown) about fabricating conventional liposomes using MF showed larger diameters of liposomes have been reported compared to the PEG liposomes in this work. The average diameter of the conventional liposomes that were prepared using DPPC and cholesterol 2:1/1:4 FRR ratio was (168 ± 4 nm). In comparison, the average diameter of the P29 and P22 PEG liposomes at a 1:4 FRR was (141 ± 10 nm). The explanation for diameter reduction is the slightly negative charge of the DSPE-PEG 2000 lipid that raises the lateral repulsion intensity, pushes the lipid bilayer to curve, and decreases the size of the liposomes consequently. Other studies in the literature confirm the same trend of a reduction in the size of the liposomes after incorporating PEG lipids.^37,38^ In a deeper look, varying liposomes’ diameter is not only about incorporating PEGylated lipids into the liposomes’ composition. Changing the ratio of the incorporated PEGylated lipids from P22 to P17 affects the liposomes̀ diameter and homogeneity. Some ratios, including P27, P19, and P17, lack stability and reproducibility from day 0, as the formulation shows nonhomogeneous results with high SD and PDI values (Figures 2 and 3). For P27, the average diameter was (193 ± 123) with a PDI average (of 0.29). For P19 and P17, the average diameter was (119 ± 109), (185 ± 105), and the PDI average was (0.29) and (0.31), respectively. The increase of PEG lipid mol % from 1.2% for P19 to 2.3% for P17 and from 0.6% for P29 to 1.7% for P27 results in the increase of the liposomes̀ diameter; the same increase of liposome diameter is consistent with other studies in the literature.^32^

The high PDI values, shown in Figure 3, and the discrepant unstable population of P27, P19, and P17 may have occurred due to the unsuitable PEG lipid mol % to the lipid ratio. It has been reported in the literature that increasing the PEG mol % to unsuitable concentrations may destabilize the lipid bilayer.^39^ Since the relationship between the PEG lipid concentration and the liposome size is not directly due to interference from other parameters, determining an optimal concentration to achieve the desired size and stability was the primary purpose. For example, Garbuzenko et al. studied the effect of increasing the DSPE-PEG2000 concentration on the liposome size. The results show that increasing the concentration from 0 to 4 mol % shows a decrease in the liposomal diameter. The increase from 4 to 8 mol % increases the liposomal diameter, and further increases >8 mol % show a reduction in liposome size.^39^ Moreover, some studies in the literature reported a potential effect of ethanol on enhancing the permeability, causing interdigitation of the membranes, and coalescing of the small liposomes.^40^ Since ethanol is used as the organic solvent for liposome preparation, a possible combined effect of ethanol and unsuitable PEG lipid concentration has destabilized the bilayer phospholipid fragment and driven them to assemble irregularly.^41^ To improve the stability of the lipid bilayer, all of the formulation is incorporated with 30–50% cholesterol to modulate the rigidity of the bilayer membrane and enhance the stability of the liposomes.^42^ Overall, the lipid ratio is not the only parameter affecting liposomes̀ diameter and homogeneity; the MF parameters, specifically FRR, have a leading role in liposomal formulation characteristics. The most optimum formulations were P29 and P22 at TFR 1 mL/mL and 1:4 FRR, showing small liposome diameter, high homogeneity (PDI < 0.25), and good stability have been determined to move forward to PX encapsulation assays.

### 3.2 Liposomal Formulation Characterization

Liposomes with small diameters (<200 nm) are usually chosen as drug carriers for more than one critical reason, such as bypassing any immunological response that may limit the efficacy of the DDS. In addition, the high surface area of the small liposomes facilitates drug release by diffusion and boosts the penetration of the DDS into the biological barriers.^43^ P29 and P22 have superior characteristics to function as a nanocarrier for PX, such as the small diameter <200 nm and high homogeneity (PDI < 0.25). The encapsulation of PX into liposomes can overcome the main limitations of PX (e.g., the low aqueous solubility and drug resistance) by enhancing their solubility and inhibiting the transporters of drug efflux in cell membranes.^44^ P29 and P22 were determined to encapsulate two concentrations of PX, 0.08 and 0.1 mg/mL, at TFR 1 mL/min/1:4 FRR to study the impact of changing the drug concentration upon the liposomes. The concentrations used have been determined based on previous reports in the literature showing that the higher solubility of PX and viable stability of liposomes reported at (3–6) mol % PX concentration.^45^ By calculating the number of moles of the used PXT con 0.08 and 0.1 mg/mL, the mol % of both concentrations were 4 and 6%, respectively. The drug-to-lipid molar ratio for P22 formulations is 1:17 for 0.08 mg/mL/1:13 for 0.1 mg/mL, and for P29 formulations, it is 1:20 for 0.08 mg/mL/1:16 for 0.1 mg/mL.

#### 3.2.1 Particle Size, PDI, and Zeta Potential

**Figure 4:**
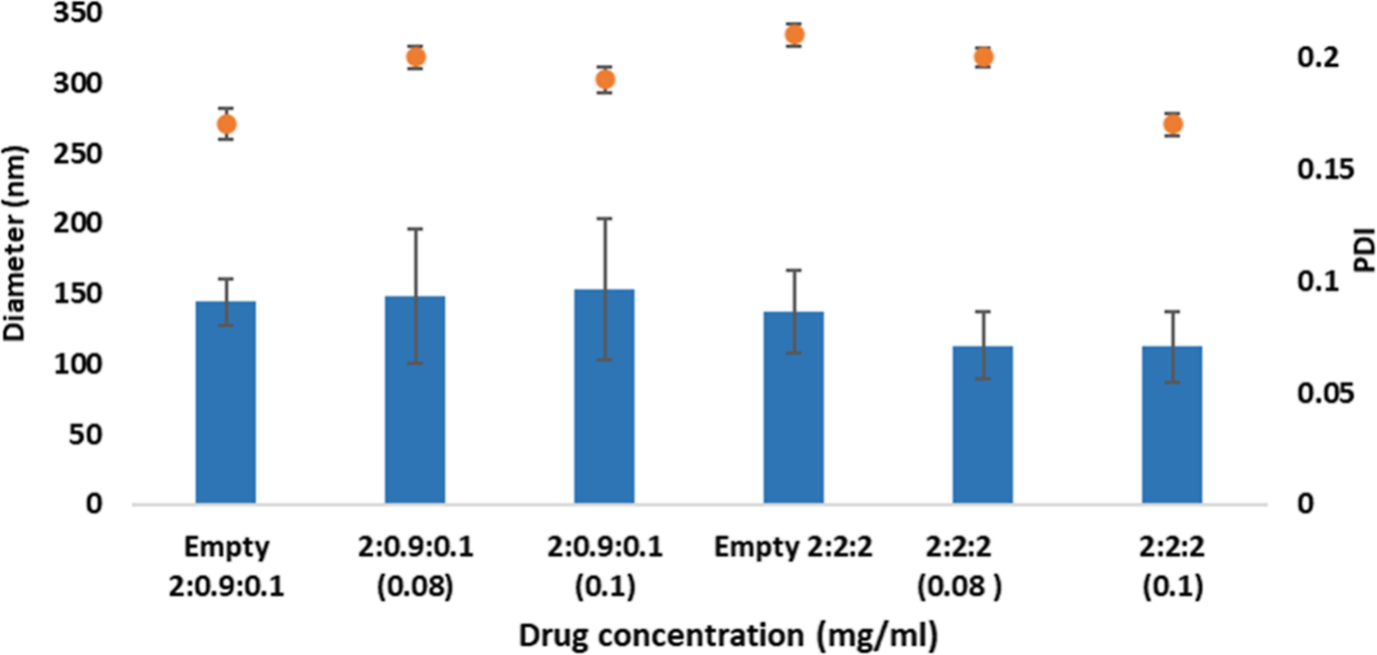
Average diameter and polydispersity of the empty and loaded P22 and P29 PEGylated liposomes.

The DLS measurements of both formulations, P29 and P22, provide a slight variation in the particle size, PDI, and Z potential. A variation in the size of the empty liposomes can be noticed, as the increase of the PEG lipid ratio from (0.6 mol %) P29 to p22 to (9 mol %), decreasing the diameter of the liposome from 144 to 137 nm, respectively (Figure 4). The PEG lipid composition can be compared since both formulation lipids ratios report a stable and homogeneous result. The PEG lipid concentration affects the PEG chains’ configuration around the surface of the liposome. At low concentrations <5 mol %, the PEG chain configuration is a mushroom-like shape (Figure 4); when the concentration is increased to >5 mol %, the configuration of the PEG chain starts to transition to a brush-like shape.^46^ By increasing the PEG lipid concentration, the PEG moieties extend and are converted gradually from a mushroom to a brush-like shape, increasing the liposome’s surface coverage.^47^ At P22, with a 9 mol % PEG lipid, the high concentration of the PEG lipid enhances the lateral repulsion of the PEG chains, which curves the lipid bilayer and reduces the vesicle size.

Various changes in liposome diameter were reported after PX encapsulation for both formulations, P22 and P29. The P29 formulation did not show a significant difference in the diameter after PX encapsulation, as the liposome diameter slightly increased at 0.08 and 0.1 mg/mL PX concentration (Figure 4). For P22, the liposome diameter decreased after encapsulating PX from 137 to 113 nm for 0.08 mg/mL and 112 nm for 0.1 mg/mL. The variation between P22 and P29 after PX encapsulation can be related to the different PEG compositions; the higher PEG concentration shows a reduction in liposome size. The size reduction of P22 is relevant to the tight packing of the hydrophobic PX molecules within the bilayer.^22^ The higher packing of PX molecules at P22 is associated with the bilayer’s compressibility; the compressibility increase reflects the dehydration of the lipid bilayer, which has a role in stabilizing the PEG liposomes and enhancing the lateral packing of acyl chains involving the PX molecules.^39^

The size uniformity of liposomes (PDI) is as important as the particle size; both factors impact the stability of the formulations and the efficacy of the drug formulation. The reported studies show that low PDI is one of the most significant physiochemical characteristics that enhance the endocytosis process of cellular uptake of liposomes, which boosts the efficacy of the DDS.^48^ Furthermore, the size and size distribution of liposomal drug formulation are considered “critical quality attributes (CQAs)” according to the FDA’s “Guidance for Industry”.^49^ A PDI of <0.25 is considered an acceptable value for liposomal drug formulation and indicates a homogeneous population.^50^ A fluctuation in the PDI values of the empty and loaded P22 and P29 is represented (Figure 4); the higher PEG concentration of P22 results in an increase in the PDI. The increase of PEG content from P29 to P22 increases the PDI from 0.17 to 0.21. The same trend of obtaining higher PDI values when the PEG concentration increases has been reported previously.^3,41^ Cheung and Al-Jamal prepared liposomal formulations by MF using DPPC lipids and different ratios of PEG lipid DSPE-PEG2000. The result shows that an increase in the mol % of DSPE-PEG200 decreases the liposomes̀ diameter and increases the PDI.^41^ After PX encapsulation, the PDI values increased for P29 from 0.17 to 0.2 for 0.08 and 0.19 for 0.1 mg/mL and decreased for P22 from 0.21 to 0.2 for 0.08 and 0.17 for 0.1 mg/mL. As shown previously in the literature, the incorporation within a phospholipid bilayer has been shown to display an element of steric hindrance upon the forming of the bilayer,^51^ which is predicted to be one of the causative factors for the change in PDI upon encapsulation. However, the PDI average for both formulations after encapsulation was ≤0.2. The promising result of PDI is owed to the unique system of mixing offered by MF. Compared to other studies using other conventional methods, such as film hydration, the PDI values increased to double after PX encapsulation.^52^

**Figure 5:**
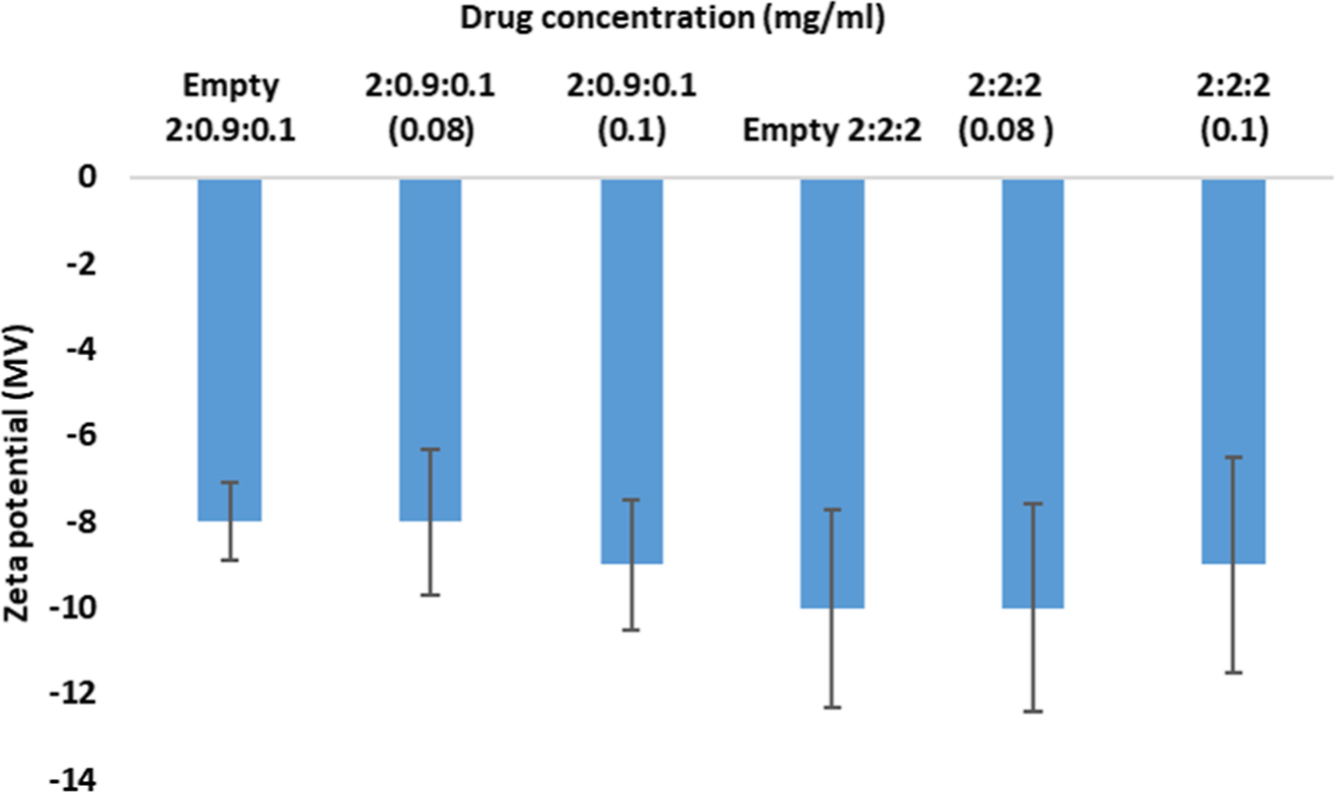
Average of the zeta potential of P29 and P22 formulations.

Zeta potentials were measured for both formulations before and after PX loading to study any changes in the electrostatic charge of liposomes after PX encapsulation. Zeta potential is a major factor affecting the liposomes’ properties, especially the stability of the formulation, as well as indicating the pharmacological interactions of the molecules.^53,54^ The empty liposomes of both formulations were slightly anionic, with −8 mV for P29 and −10 mV for P22. In general, conventional liposomes have a neutral to slightly anionic charge due to the orientation of the negative phosphate group toward the surface of the liposomes instead of the choline group. The hypothesis assumes that the partially hydrophobic nature of the choline group due to the methyl groups at the nitrogen end makes the choline group oriented toward the interphase of liposomes to avoid contact with the aqueous phase. The PEGylation of liposomes in this project provides more anionic liposomes compared to our previous work results of preparing conventional DPPC liposomes with a −7 mV charge.^22^ The greater negative charge of the PEG liposomes is related to the slightly negative charge of the DSPE-PEG2000. After encapsulation, the liposomes become more anionic for P22 and have a negligible effect on P29 (Figure 5). The decrease in zeta potential gives an advantage of enhancing the stability of the loaded liposomes due to the increase of repulsion forces between liposomes, which prevents aggregation.^55^ Moreover, the neutral and anionic liposomes have prolonged blood circulation and a higher ability for passive diffusion into the tissues.^56^

#### 3.2.2 Stability Study

**Figure 6:**
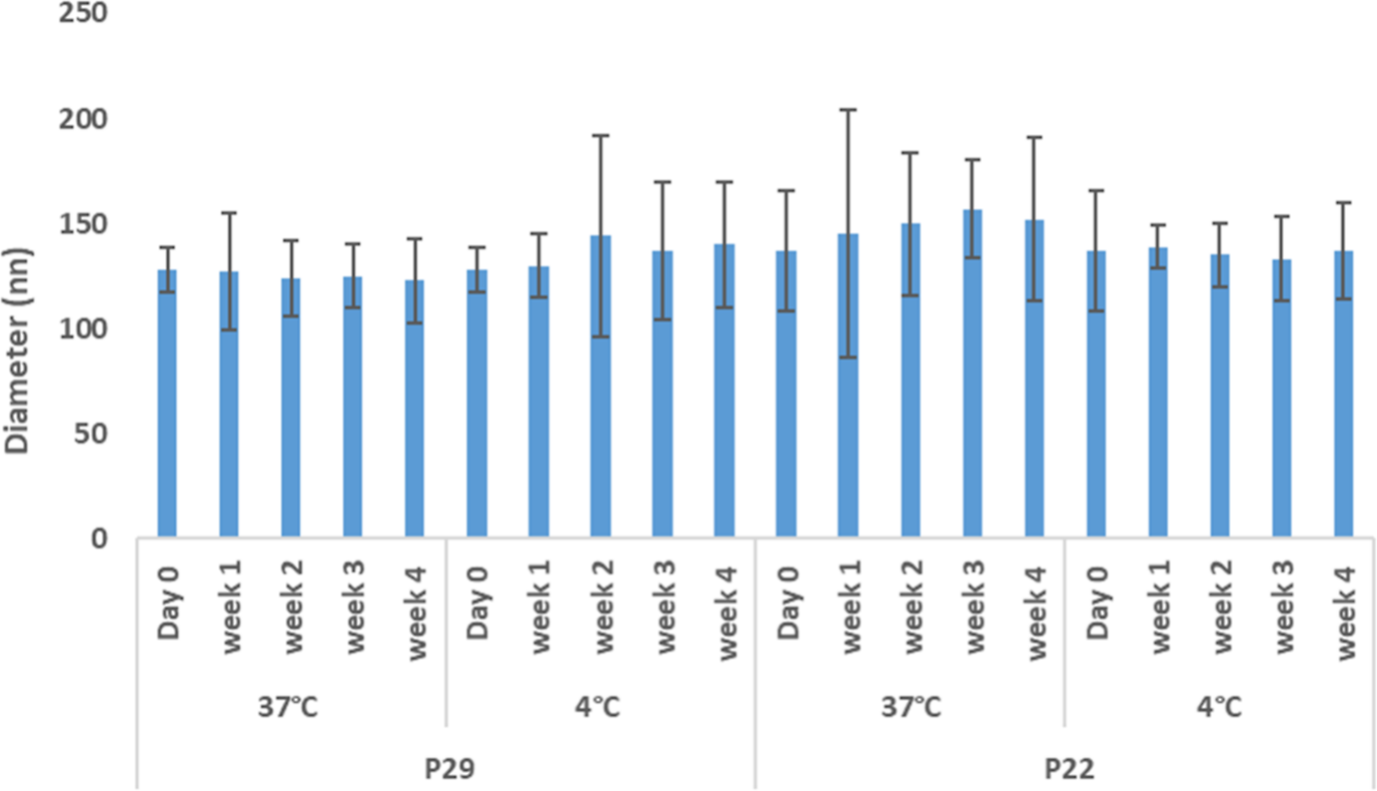
Stability study of both P22 and P29 empty PEGylated liposomes.

The physical stability of P29 and P22 is measured over 4 weeks to ensure a low aggregation incidence and appropriate liposomal formulation homogeneity. Liposome aggregation can lead to premature drug release and variation in delivery efficiency.^57^ The stability study for empty liposomes was performed first as a control to test the physical stability of the liposomes as carriers (Figure 6). The stability study’s results reported that most of the formulation particles̀ size increased at 37 °C compared to 4 °C, which keeps the particle size relatively constant. By comparing the lipid ratios P29 and P22 stability, the former shows more comparable particle size during the 4 weeks of stability at 37 °C. The PDI results support that the PDI of empty P29 increased at 37 °C from 0.17 to 0.2 and from 0.21 to 0.23 for the empty P22. Both formulations show high stability at 4 °C. The high stability of the PEG liposomes supports other previous results in the literature regarding the enhanced stability of liposomes after PEG lipid incorporation. Several studies reported the impact of the PEG lipid on avoiding liposome aggregation, which enhances their physical stability.^58−60^ The increase of steric hindrance after the addition of PEG lipid chains has the main role in enhancing stability.^59^ The stability study of liposomes after PX encapsulation has been performed to ensure the physical stability of the DDS (Figures S1 and S2). The increase in liposome size during the stability study might have occurred due to the orientation of the polar headgroup to compensate for the high packing imposed by the lateral interactions of the hydrocarbon chains after PX encapsulation.^61,62^ The same trend of increasing liposomes after the incubation at 37 °C has been reported in the literature.^61,63^

#### 3.2.3 FTIR Results

The main aim of performing FTIR analysis is to study the effect of liposomal modification with PEGylated lipids on the vibration of the chemical bonds and to determine if any newly generated bonds have emerged. The resulting peaks show specific functional groups (Figure S3), including the O–H bond that appeared at the 3218–3349 cm^–1^ due to a primary alcohol group. The possible reason for the existence of the O–H peak is the presence of ethanol traces that have been used during formulation manufacture. The C–H bond peaks detected appearance at 2850–2958 cm^–1^, which indicates stretching in the symmetric region of the alkane chain. A medium peak appeared at 1636 cm^–1^ and was revealed to be the amine group NH_2,_ which is a main component of DSPE-PEG 2000. Compared to FTIR spectra of the conventional liposomes that were formulated in our previous work (result not shown), this peak was not present, which confirms the PEGylation of the liposomes.^22^ The C–H bond of alkane peaks was observed with varying shapes at the range 2850–2958 cm^–1^, which indicates C–H stretching in the symmetric region of the alkane chain.^64^ A sharp peak appeared at 1044 cm^–1^ for the P–O bond in the PO_4_ group of phospholipid. The detection of the peak in this range indicates symmetric stretching vibration in the phosphate group.^65^ After PX encapsulation, the region of the symmetric C–H starching vibration shifted from 2978 to 2983 cm^–1^. The region of symmetric C–H stretching vibration is measured by the number of gauche conformers in the hydrocarbon chains; the alteration in this region indicates an increase of the gauche conformers and changes in the arrangement of the hydrocarbon chains, which confirms PX incorporation within the bilayer.^64^ Additionally, a shift in the N–H symmetric region was detected after PX encapsulation; the peak shifts from 1636 to 1638 cm^–1^. Any minor change in the symmetric region can be critical due to its sensitivity to any mobility or conformational change within the chains.^66^

#### 3.2.4 Atomic Force Microscopy

**Figure 7:**
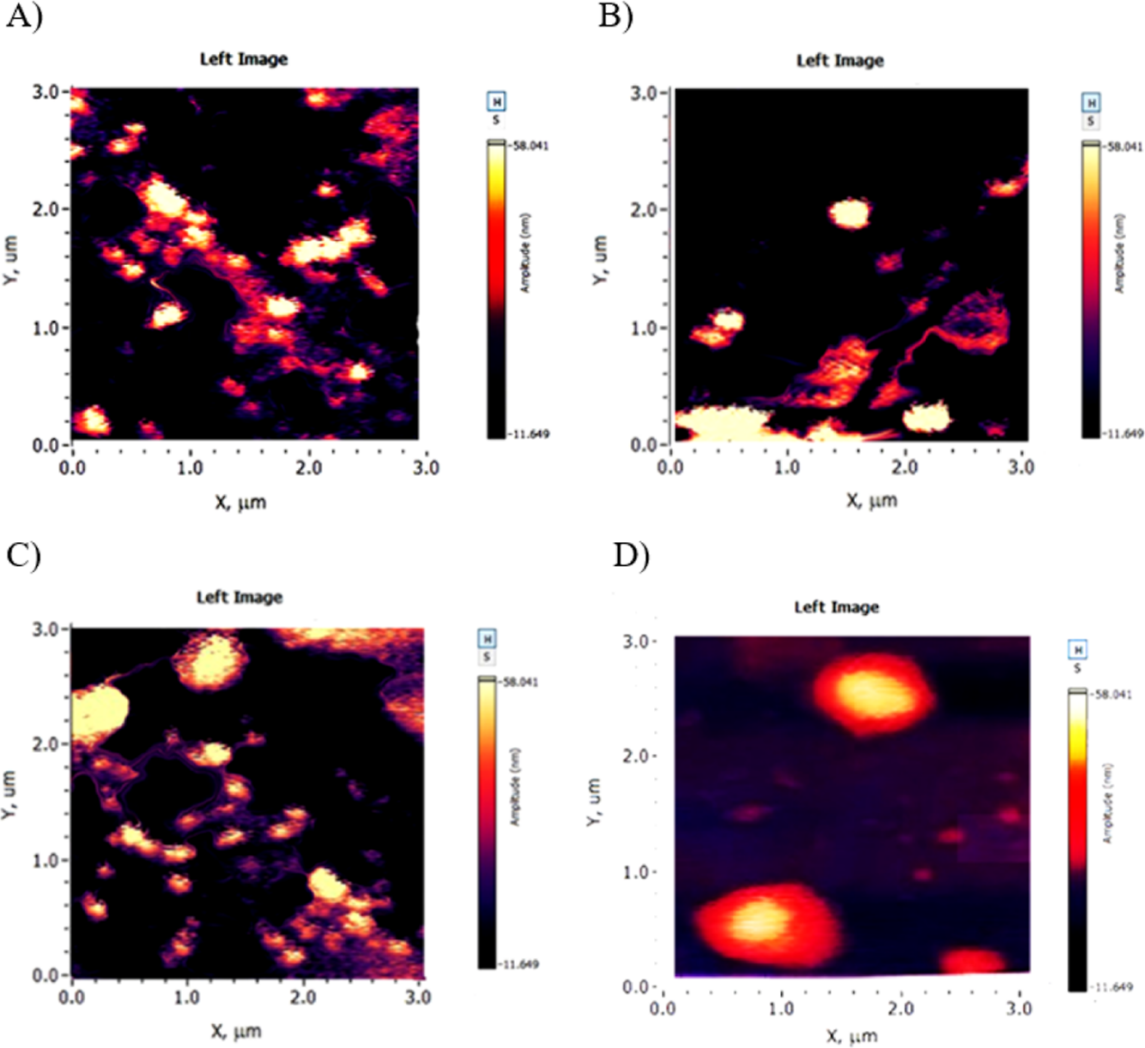
AFM images for (A) empty P29 formulation, (B) loaded P29 formulation, (C) empty P22 formulation, and (D) loaded P22 formulation.

The AFM images give a visual description of the morphological shape of the liposomes. The images have been taken for the empty and loaded P22 and P29 with 0.1 mg/mL PX (Figure 7). The images generally show semicircular and uniform shapes for empty liposomes for P29 and P22. However, the nonuniformed shapes represented in the images might be due to the drying step affecting the liposome’s shapes and uniformity.^23^ After PX encapsulation, the morphological shape of liposomes changes to be more circular and uniform, which supports the DLS results of decreasing the particle size and PDI after PX incorporation. The enhanced uniformity of liposomes after PX incorporation can be explained by the tight packing of PX within the bilayer and the enhancement of lateral packing of the complete acyl chains.^61^

### 3.3 Encapsulation Efficiency

**Figure 8:**
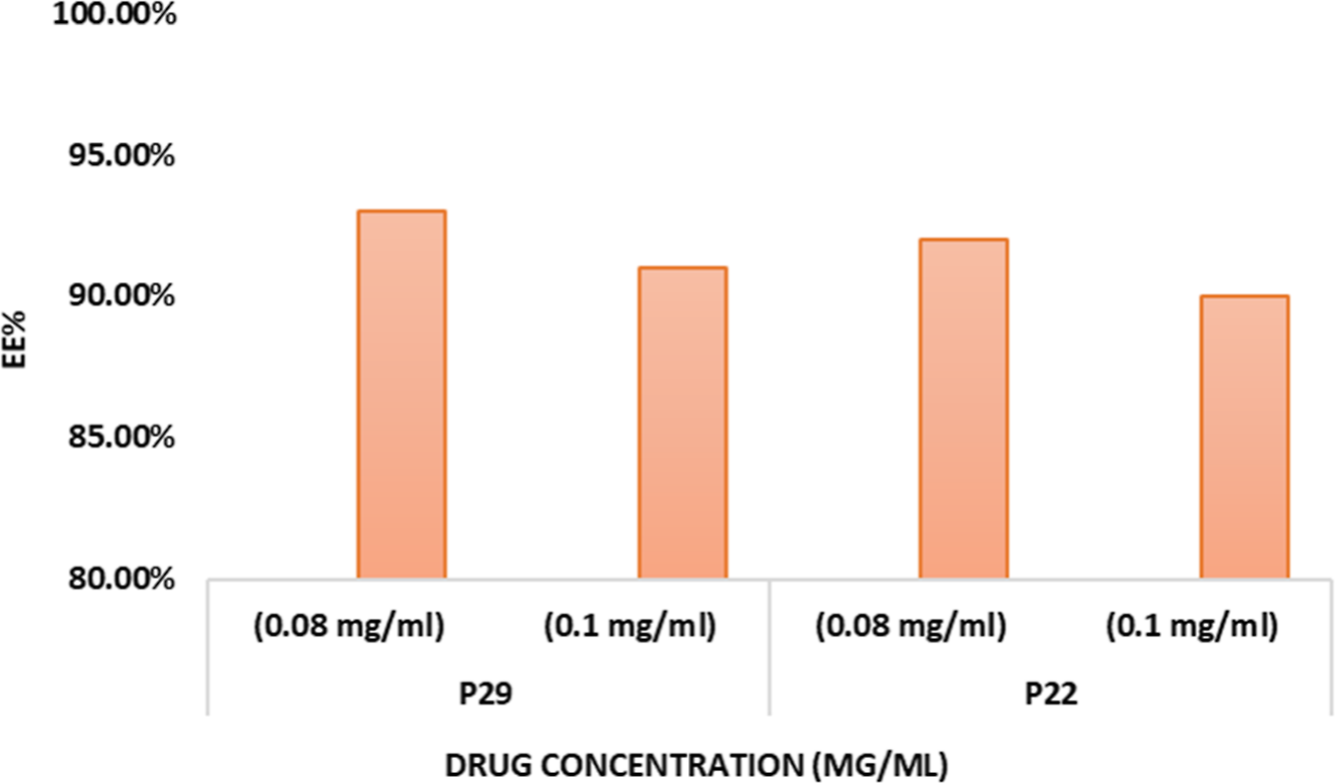
EE % of P22 and P29 at 0.08 and 0.1 mg/mL.

The EE % of P22 and P29 at the two different PX concentrations was higher than 90% (Figure 8). The use of the MF system as the formulating method has a crucial role in increasing the EE % of the formulation compared to other conventional methods, such as film hydration. Several studies in the literature show the limited capability of conventional methods to achieve high EE %; for example, the EE % of thin-film hydration and extrusion using PX was less than 50%.^15,31^ Multiple other parameters can affect the EE %, including the choice of lipid. The usage of specific amounts of cholesterol (30–50%) shows a positive impact on the EE % due to the hydrophobic nature of cholesterol, which may consequently increase the hydrophobicity of the bilayer membrane and enhance the entrapment of the hydrophobic API.^42^ Also, a slight increase in the EE % (2–3%) was reported after PEG lipid incorporation. The addition of PEG lipids to the liposomes’ composition affected the EE % positively; the EE % of P29 with 0.1 mass ratio of DSPE-PEG2000 was 93% compared to the conventional liposomes that were formulated previously with the same lipids.^22^ Other studies reported an increase in the EE % after PEG lipid incorporation.^14,67^ Tsermentseli et al. made a comparative study between conventional liposomes and PEGylated liposomes as a carrier. The researchers formulated conventional liposomes using phospholipids, cholesterol, and PEGylated liposomes by incorporating DSPE-PEG2000. The results show how the addition of different mass ratios of DSPE-PEG2000 increased the EE % by 12–13%.^14^ The increase in EE % might be due to the presence of PEG chains on the outer surface of the lipid bilayer, which can enhance the entrapment of the PX into the bilayers.^63,68^ However, the results represent a mild reduction in the EE % for the formulation with a higher PEG concentration (P22) compared to (P29) at a specific drug concentration. This reduction might explain that the increasing of PEG lipids to high concentrations may decrease the lamellae of the liposomes due to the steric repulsion of the large head groups.^3^ The variation in drug concentration represents a minor impact on the EE % for both P29 and P22; the EE % of P29 at 0.08 and 0.1 mg/mL was 93 and 91%, respectively. For P22, at 0.08 and 0.1 mg/mL, the EE % was 92 and 90%, respectively. However, the PX concentration between 3 and 6 mol % can achieve the best solubility and EE %, based on previous results in the literature.^45^

### 3.4 In Vitro Drug Release

**Figure 9:**
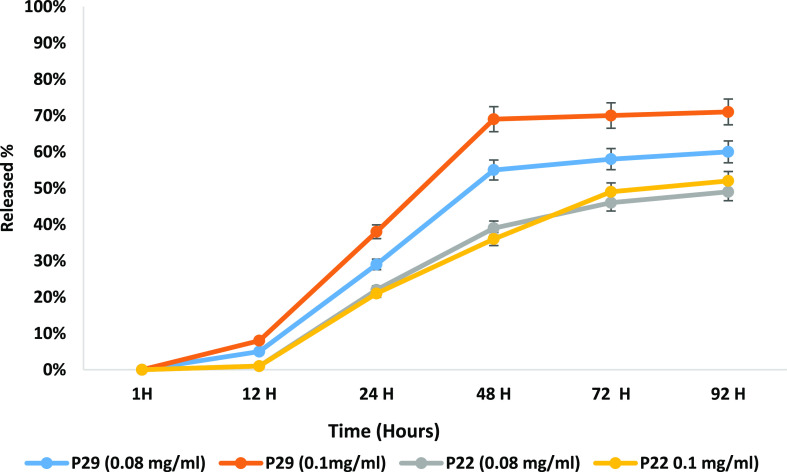
Drug release profile for P29 and P22 at 0.08 and 0.1 mg/mL concentrations.

The release profiles of both formulations P22 and P29 were tested in vitro for 92H (Figure 9). The results show variation in the release rate and the % of the released drug; P29 achieved a higher released drug % with a faster release rate. The release of PX from P29 starts at 12H and reaches an average of 70% of the released drug for both concentrations after 72H. In contrast, the release of P22 starts after 12H and reaches an average of 48% released drug for both concentrations after 48H. The steady state for P29 formulations was achieved at 48H and for P22 formulation at 72H, which are the times that the drug concentration becomes consistent. The study was performed for 92H to confirm the steady-state time. The release profile for both formulations displayed delayed-release characteristics as the PX release starts at or after 12 h. The delayed release of the PEGylated formulation is due to the presence of PEG coating on the surface, which renders the API release slow over time in a sustained way.^67^ The delayed release of the API can be an advantage in avoiding the premature release of PX and limiting the collateral toxicity for healthy tissues.^69^ The increase in the PEG lipid ratio increases liposome rigidity, one of the main parameters affecting drug release.^70^ The higher PEG concentration in the P22 formulation increased the liposomes’ rigidity and showed a slower release. The reported results for P22 show that the increase in the drug concentration from 0.08 to 0.1 mg/mL had a minor effect on the released drug percentage. This may happen due to the higher PEG lipids ratio; the PEGylated liposomes have more compressed membranes and more space inside the structure of the liposome.^70,71^ It can be summarized that the higher the rigidity of the bilayer, the slower the release of the drug. Other previous studies in the literature reported the same slower release with the incorporation of PEG lipids.^72−74^ Moreover, these results support our previous results of PX release from conventional liposomes (results not shown), which shows a faster release rate and reached a steady state at 48 h.^22^

## 4 Conclusions

The conducted experiments investigate the impact of changing the FRR and incorporating different DSPE-PEG2000 ratios into liposome composition on the particle size, PDI, EE %, release profile, and stability. The alteration of FRR impacts the liposome size and PDI significantly; increasing the FRR from 1:4 to 1:6 decreases the particle size and increases the PDI of empty PEG liposomes. The FRR of 1:4 was the optimum ratio to produce controlled-size liposomes with low PDI. The capability of MF to control the FRR and TFR has a primary role in enhancing the final liposome quality. MF system shows high efficiency in formulating PEG liposomes with controlled size, high homogeneity, and EE %. The significance of MFs̀ high mixing quality is shown by the improvement in liposome size and PDI compared to other conventional methods.^52^ Moreover, automated and computerized systems offer more advantages than traditional methods, such as being a time-saving one-step process. PEG lipid ratio has highly affected the stability of the liposomes, as a result showing that unsuitable PEG ratios provide unstable and unreproducible liposomes. P29 and P22 were the most suitable ratios for formulating stable PX-loaded liposomes. The increase of PEG lipid from the P29 to P22 ratio results in a decrease of the particle size, an increase of the PDI, a slight reduction of the EE %, and more sustained release. Overall, PX presents high levels of packing in both P29 and P22. The PEGylated liposomal formulation seems to be promising for the future.

## Supporting Information Available

Stability study of P29, stability study of P22, and FTIR spectra of empty and loaded liposomes (PDF)
