# Supplementary material for: Synthesis and Characterization of Paclitaxel-Loaded PEGylated Liposomes by the Microfluidics Method
Source: Mol Pharm. 2023 Nov 6;20(12):6184–96. doi: 10.1021/acs.molpharmaceut.3c00596 (PMC10698720; doi:10.1021/acs.molpharmaceut.3c00596)
Supplement: Supplementary file 1 — mp3c00596_si_001.pdf [file mp3c00596_si_001.pdf]

Synthesis and Characterization of Paclitaxel-Loaded PEGylated Liposomes by Microfluidics Method

Eman Jaradat[1], Edward Weaver[1], Adam Meziane[2], Dimitrios A. Lamprou[1*]

[1]School of Pharmacy, Queen's University Belfast, 97 Lisburn Road, Belfast BT9 7BL, UK

[2]Fluigent, 94270 Le Kremlin-Bicêtre, France

Supplementary information

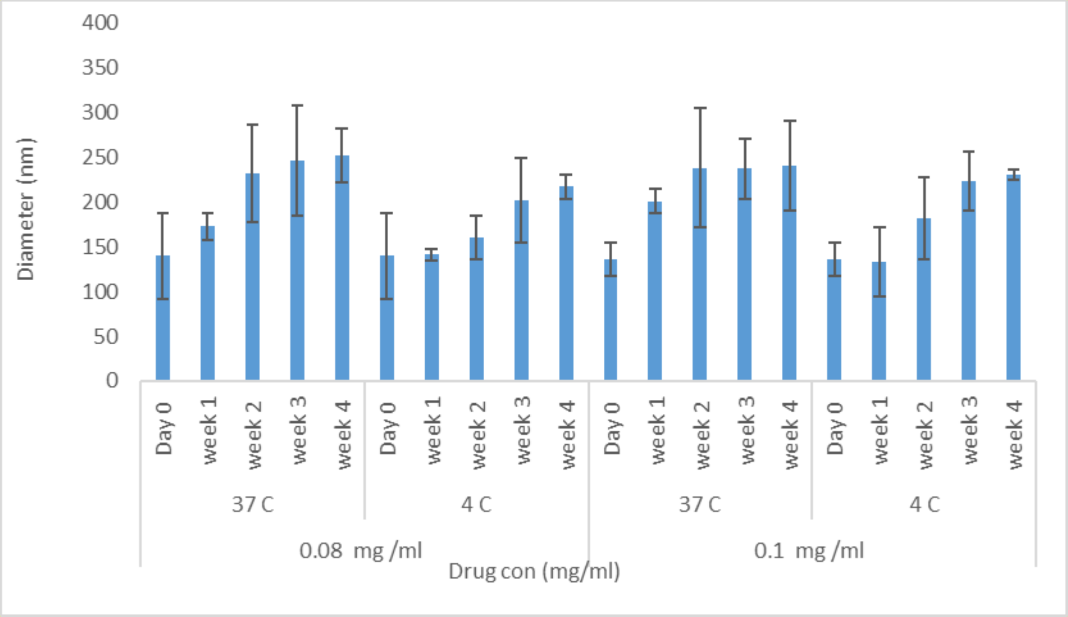

Figure S1. stability study for P29 PXT loaded liposomes at 0.08 and 0.1 mg/ml concentrations

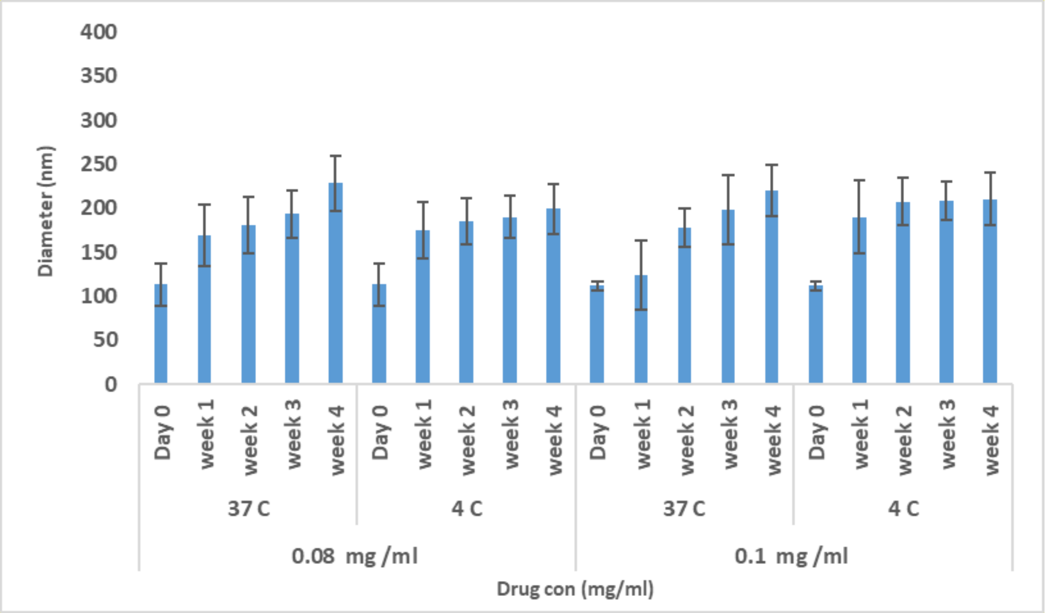

Figure S2. stability study for P22 PXT loaded liposomes at 0.08 and 0.1 mg/ml concentrations.

A)

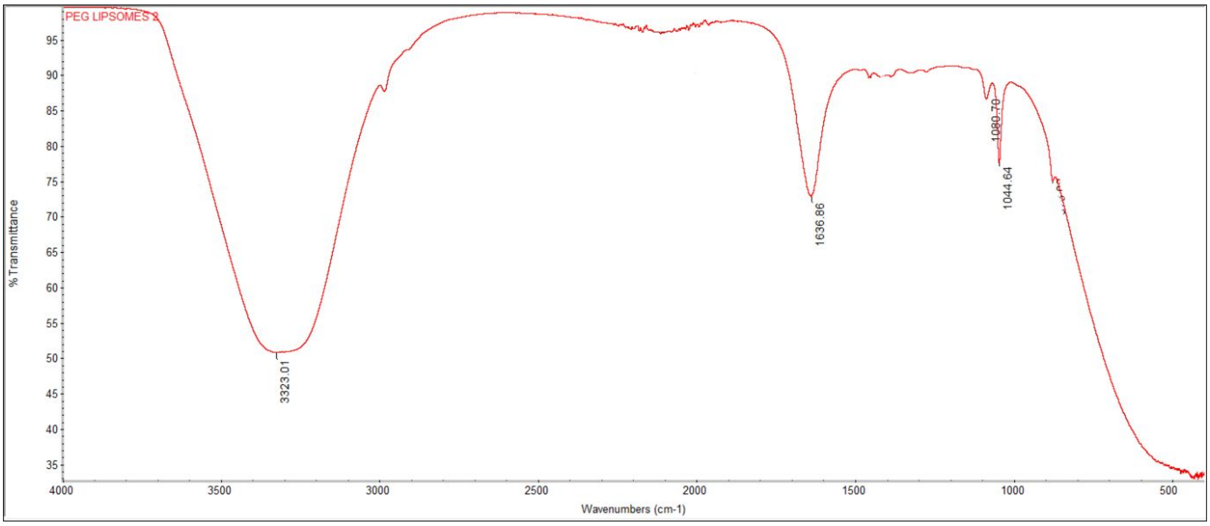

B)

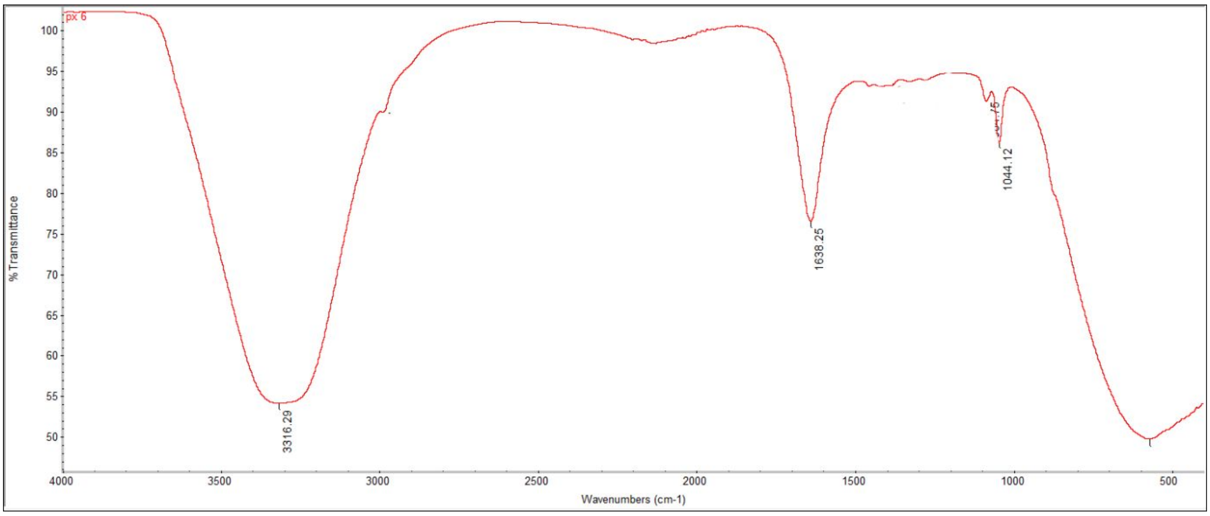

Figure S3. The FTIR spectra obtained for: (A) empty PEGylated liposomes and (B) loaded PEGylated liposomes.
